# Alzheimer’s disease profiled by fluid and imaging markers: tau PET best predicts cognitive decline

**DOI:** 10.1038/s41380-021-01263-2

**Published:** 2021-10-01

**Authors:** Marco Bucci, Konstantinos Chiotis, Agneta Nordberg, for the Alzheimer’s Disease Neuroimaging Initiative

**Affiliations:** 1 Division of Clinical Geriatrics, Center for Alzheimer Research, Department of Neurobiology, Care Sciences and Society, Karolinska Institutet, Stockholm, Sweden; 2 Department of Neurology, Karolinska University Hospital, Stockholm, Sweden; 3 Theme Aging, Karolinska University Hospital, Stockholm, Sweden

**Keywords:** Prognostic markers, Diagnostic markers

## Abstract

For early detection of Alzheimer’s disease, it is important to find biomarkers with predictive value for disease progression and clinical manifestations, such as cognitive decline. Individuals can now be profiled based on their biomarker status for Aβ42 (A) or tau (T) deposition and neurodegeneration (N). The aim of this study was to compare the cerebrospinal fluid (CSF) and imaging (PET/MR) biomarkers in each ATN category and to assess their ability to predict longitudinal cognitive decline. A subset of 282 patients, who had had at the same time PET investigations with amyloid-β and tau tracers, CSF sampling, and structural MRI (18% within 13 months), was selected from the ADNI dataset. The participants were grouped by clinical diagnosis at that time: cognitively normal, subjective memory concern, early or late mild cognitive impairment, or AD. Agreement between CSF (amyloid-β-1-42(A), phosphorylated-Tau181(T), total-Tau(N)), and imaging (amyloid-β PET (florbetaben and florbetapir)(A), tau PET (flortaucipir)(T), hippocampal volume (MRI)(N)) positivity in ATN was assessed with Cohen’s Kappa. Linear mixed-effects models were used to predict decline in the episodic memory. There was moderate agreement between PET and CSF for A biomarkers (Kappa = 0.39–0.71), while only fair agreement for T biomarkers (Kappa ≤ 0.40, except AD) and discordance for N biomarkers across all groups (Kappa ≤ 0.14) was found. Baseline PET tau predicted longitudinal decline in episodic memory irrespective of CSF p-Tau181 positivity (*p* ≤ 0.02). Baseline PET tau and amyloid-β predicted decline in episodic memory (*p* ≤ 0.0001), but isolated PET amyloid-β did not. Isolated PET Tau positivity was only observed in 2 participants (0.71% of the sample). While results for amyloid-β were similar using CSF or imaging, CSF and imaging results for tau and neurodegeneration were not interchangeable. PET tau positivity was superior to CSF p-Tau181 and PET amyloid-β in predicting cognitive decline in the AD continuum within 3 years of follow-up.

## Introduction

The rapid development of several biomarkers for Alzheimer’s disease (AD) during recent years has allowed in vivo tracking of the pathological components of the disease. Some of these biomarkers were designed to target AD-specific changes, such as the deposition of amyloid-β (Aβ) and tau, while others target downstream neurodegeneration. Aβ- and tau-specific positron emission tomography (PET) ligands allow in vivo evaluation of the Aβ and tau components of brain pathology, respectively, and the same is claimed to be possible for the fluid biomarkers such as cerebrospinal fluid (CSF) levels of Aβ42 and tau phosphorylated at Thr181 (pTau). A series of biomarkers have also been proposed for downstream neurodegeneration; these include brain atrophy measured by magnetic resonance imaging (MRI), total tau levels in the CSF (tTau), and glucose metabolism changes as assessed by PET [1]. Although the different modalities (PET/MRI imaging, CSF) for each of the Aβ, tau and neurodegeneration (ATN) components have been approved for clinical use and are considered interchangeable by some expert consensus groups [1], concordance between the imaging and CSF biomarkers has recently been questioned [2, 3]. A recent paper provides recommendation for the use of AD biomarkers in the clinical setting for AD diagnosis. [4,5] However, guidelines for the use of specific biomarkers across or within the ATN components, which will aid the early and accurate clinical prognostic assessment and treatment of patients with cognitive complaints, are still lacking. A common strategy to assess the utility of the AD biomarkers and ATN profiles is to look at their prognostic value on the cognitive decline. Delmotte at al found that ATN profiling based on CSF has a clinically relevant prognostic value for the course of cognitive decline with 3 years follow-up [6]. In longitudinal studies such as this one, it would also be of interest to evaluate ATN profiling based on imaging biomarkers, and further to compare it to profiling with CSF biomarkers. Other groups have partly addressed this, by focusing on profiling ATN-T (CSF and PET Tau dichotomization) only and investigated mainly cross-sectionally [3, 7] or retrospectively [8]. One group has looked at ATN profiling with both imaging and CSF biomarkers longitudinally [2] (with MMSE as cognitive measure), but more longitudinal studies with different cohorts, bigger sample size, and better outcome measures are needed.

In this study, we characterized participants in the Alzheimer’s Disease Neuroimaging Initiative (ADNI) according to their cognition status, ranging from normal to dementia, compared to their ATN component status (positive/negative biomarkers). Our aim was twofold. Firstly, we wanted to assess the level of agreement/concordance between the imaging and CSF biomarkers across the ATN components. Secondly, we aimed to evaluate which of the investigated biomarkers best predicted subsequent cognitive decline in episodic memory function, the cognitive domain that is typically affected at the earliest stages of AD.

## Material and methods

### Subjects

The data were downloaded from the ADNI website (http://adni.loni.usc.edu) on 09/04/2021. Additional information is found at www.adni-info.org. ADNI was launched in 2003 for testing whether neuroimaging biomarkers in combination with clinical, biological, and neuropsychological markers could measure progression in AD spectrum.

All research complied with the ethical principles of the Declaration of Helsinki. Written informed consent was obtained for all participants, and study procedures were approved by the institutional review board at each of the participating centers.

ADNI participants who had at least one PET ^18^F-flortaucipir (FTP) assessment were initially selected (*n* = 764). However, only participants who had had CSF samples taken for Aβ42, tTau, and pTau assessment, had had PET Aβ ^18^F-florbetapir (FBP) or ^18^F-florbetaben (FBB) assessment on the same visit code as the baseline PET tau, and had also had a structural T1 MRI scan within 13 months of baseline were finally included (*n* = 282). For 51/282 (18%) subjects, the MRI scans were obtained later than the baseline visit (mean difference in days ± sd:349 ± 42, range:161–394).

### Clinical diagnosis

The subjects were grouped according to their clinical diagnosis at baseline into the following groups: Cognitively normal (CN, *n* = 90), Subjective memory concern (SMC, *n* = 91), Early/Late MCI (EMCI, *n* = 41; LMCI, *n* = 36) and AD dementia (*n* = 24).

CN subjects (by clinical assessment) were classified as SMC if they had a cognitive change index score (first 12 questionnaire items) ≥16 according to ADNI2 guidelines [10].

The distinction between EMCI and LMCI was based on Clinical dementia rating, Mini-Mental State Examination and education-adjusted severity of episodic memory impairment (measured by the Logical Memory II recall test), following ADNI2 thresholds [11].

### PET imaging biomarkers

The files containing the regional PET uptake data for Aβ (FBP; FBB) and tau (FTP) were downloaded from the ADNI website (UCBERKELEYAV45_/UCBERKELEYFBB_/ UCBERKELEYAV1451_01_14_2021.csv). These data had been pre-processed using the ADNI pipeline [12]. PET Aβ positivity (A+) was considered if the global SUVRs of FBP and FBB PET results were ≥1.11 and 1.08, respectively [7, 12]. For FTP PET, we considered tracer binding in a weighted composite region comprising the bilateral entorhinal cortex, amygdala, fusiform gyrus, and inferior and middle temporal cortices (i.e., the temporal meta-ROI, or T1 ROI) [7, 8]. The inferior temporal cortex (T2) and entorhinal (T3) ROI were also assessed according to methods previously described [13]. PET tau positivity (T+) was considered if the SUVRs were ≥1.37 [8], 1.31 and 1.39 [13] for the T1, T2, and T3 ROIs, respectively.

### CSF biomarkers

Lumbar punctures were carried out to obtain CSF samples as described in the ADNI procedures manual (http://www.adni-info.org/). The concentrations of Aβ42, pTau, and tTau in CSF were obtained from the ADNI depository (UPENNBIOMK9_04_19_17.csv, UPENNBIOMK10_07_29_19.csv, UPENNMSMSABETA2CRM.csv, UPENNBIOMKADNIDIAN2017.csv); these were measured using Elecsys immunoassays on a cobase 601 analyzer, as previously described [14].

CSF A+ was based on CSF Aβ42 levels with a cutoff value of ≤880 pg/mL [15]. For assessing CSF T+, we used pTau, which is thought generally to reflect tau pathology; the cutoff value used was ≥26.64 pg/mL as previously published in a similar population [8]. tTau is considered to be a more general marker of neurodegeneration; the cutoff value for N+ was ≥300 pg/mL, as previously validated in the ADNI population [16].

### MRI biomarkers

Pre-processed data concerning volumetric and cortical thickness measures were also downloaded from the ADNI website (UCSFFSX6_02_05_20.csv). The primary MRI measure was the FreeSurfer (version 6.0)-derived hippocampal volume (HV) after adjusting (HVa) for intracranial volume. The measure is a proxy for neurodegeneration that correlates well with learning and memory cross-sectionally and longitudinally [17]. The calculations and the cutoff value of <6723 mm^3^ (N+) have been described previously [18]. We also evaluated an additional MRI biomarker: the temporal lobe cortical thickness composite ROI comprising the entorhinal, inferior and middle temporal, and fusiform ROIs, with a cutoff value of <2.67 mm (N+) [19].

### Cognitive measures

The ADNI (episodic) composite memory score has been validated as a reliable metric for cognitive change in the ADNI cohort [9]. The scores were precomputed via structural equation modeling in the ADNI repository (UWNPSYCHSUM_03_09_21.csv).

### Statistical analysis

The concordance of the biomarkers across the ATN framework was evaluated in a descriptive manner, including use of the Cohen’s kappa coefficient. Cohen’s kappa statistics measure inter-rater agreement for categorical items. Cohen’s kappa coefficients are reported with 95% confidence intervals and values >0.40 indicate a relevant level of agreement [20]. The prevalence of AT and ATN profiles have been visualized with barplots, Sankey (R library:”networkD3”) and Sunburst (R library: “plotly”) graphs.

The correlations between the different biomarkers were assessed with the use of the Spearman’s rho correlation coefficient (Rho) (*p* < 0.05, not adjusted for multiple comparisons).

### Longitudinal data analysis for cognition score changes

Linear mixed-effects models (LMMs) were built for assessing the effect of biomarker status in predicting prospective cognitive decline. The longitudinal ADNI (episodic) memory composite score (ADNI_MEM) was used as the outcome variable; the ADNI-MEM follow-up datapoints were predominantly distributed around 12 and 24 months and to assure that the follow-up across the subjects was homogeneous, and to exclude outliers, in our sample we limited the follow-up interval to 36 months (only 12/527 observations available were excluded), only subjects with at least two ADNI_MEM measures were included (*n* = 213, 22 ± 7.1 FU months mean ± SD, max 35.2). The biomarker profiles were modeled as factors in two sets of models for assessing: (1) the effect of concordance/discordance of CSF and PET within the same biomarker class (amyloid or tau); and (2) the effect of concordance/discordance of the two biomarkers class (A/T) within CSF or PET. Additional details on the models are reported in the Supplementary Information (SI). Ten models with different combinations of variables were tested and the model with the lowest Bayesian Information Criterion (BIC) was chosen. Tests of significance using LMMs were performed with *P* ≤ 0.05. In the longitudinal analyses, we evaluated if a particular group had difference in slope (faster decline) in the longitudinal memory follow-up compared to the reference group (-/-). All statistical analyses were performed using R software/environment (version 4.0.2) [21]. For the LMM analysis, we used the “lmerTest” and “lme4” R packages, and “leveneTest” function from “car” package to assess the homoscedasticity (equal variances between groups).

## Results

### Sample characteristics

Table 1 show the general characteristics of the study population grouped by baseline diagnosis and by cognitive impairment, respectively. In addition, Supplementary Table 1 shows the CSF- and imaging-based biomarker levels across diagnostic groups.

**Table 1:** General subject characteristics.

	CN (*N* = 90)	SMC (*N* = 91)	EMCI (*N* = 41)	LMCI (*N* = 36)	AD (*N* = 24)	Total (*N* = 282)	*p* value
Age, yrs							0.222^e^
Mean (SD)	72.9 (7.4)	71.2 (6.3)	72.7 (7.7)	74.1 (8.1)	74.2 (9.8)	72.6 (7.5)	
Range	56.5–91.5	57.1–90.4	57.8–88.1	55.9–88.4	55.5–89.2	55.5–91.5	
Sex							0.003^f^
Male	35 (38.9%)	32 (35.2%)	23 (56.1%)	21 (58.3%)	17 (70.8%)	128 (45.4%)	
Female	55 (61.1%)	59 (64.8%)	18 (43.9%)	15 (41.7%)	7 (29.2%)	154 (54.6%)	
Education, yrs							0.065^e^
Mean (SD)	17.2 (2.2)	16.7 (2.1)	16.2 (2.9)	16.0 (2.4)^a^	16.6 (2.7)	16.7 (2.4)	
Range	11.0–20.0	12.0–20.0	12.0–20.0	10.0–20.0	12.0–20.0	10.0–20.0	
APOE4 carrier							0.034^f^
Missing (*n*)	0	1	0	0	0	1	
No	63 (70.0%)	52 (57.8%)	27 (65.9%)	19 (52.8%)	9 (37.5%)	170 (60.5%)	
Yes	27 (30.0%)	38 (42.2%)	14 (34.1%)	17 (47.2%)	15 (62.5%)	111 (39.5%)	
MMSE							<0.001^e^
Mean (SD)	29.0 (1.2)	29.3 (1.0)	28.3 (1.4)^b^	27.2 (2.3)^a.,b.,c^	23.2 (2.7)^a.,b.,c.,d.^	28.3 (2.3)	
Range	25.0–30.0	26.0–30.0	25.0–30.0	19.0–30.0	17.0–29.0	17.0–30.0	
CDR							<0.001^e^
Mean (SD)	0.0 (0.1)	0.0 (0.0)	0.5 (0.1)^a.,b.^	0.5 (0.1)^a.,b.^	0.8 (0.3)^a.,b.,c.,d.^	0.2 (0.3)	
Range	0.0–0.5	0.0–0.0	0.0–0.5	0.0–0.5	0.5–1.0	0.0–1.0	
ADNI memory composite score							<0.001^e^
Mean (SD)	1.1 (0.5)	1.0 (0.6)	0.5 (0.4)^a.,b.^	0.1 (0.5)^a.,b.,c^	−0.7 (0.6)^a.,b.,c.,d.^	0.7 (0.7)	
Range	−0.2 −2.7	−0.2 −2.3	−0.3 −1.4	−1.0 −1.2	−1.6 −0.5	−1.6 −2.7	
Follow up time interval, months							<0.001^e^
Missing (*n*)	30	25	5	1	6	69	
Mean (SD)	23.6 (6.6)	24.6 (5.2)	19.4 (8.5)	17.8 (7.0)^b^	20.2 (7.2)	22.0 (7.1)	
Range	0.0–33.7	11.9–32.0	0.0–35.2	4.1–31.0	10.4–30.4	0.0–35.2	

^a,b,c,d^Denote a significant difference from CN, SMC, EMCI and LMCI, respectively, with Tukey Post Hoc. (*p* < 0.05).

^a.,b.,c.,d.^Denote a significant difference from CN, SMC, EMCI and LMCI, respectively, with Tukey Post Hoc. (*p* < 0.001).

*AD* Alzheimer’s disease, *CDR* clinical dementia rating, *CN* cognitively normal, *EMCI* early MCI, *LMCI* late MCI, *MCI* mild cognitive impairment, *MMSE* mini-mental state examination, *SMC* subjective memory concern.

^e^Linear Model ANOVA.

^f^Pearson’s Chi-squared test.

### AT profiles with CSF and imaging biomarkers

**Fig. 1:**
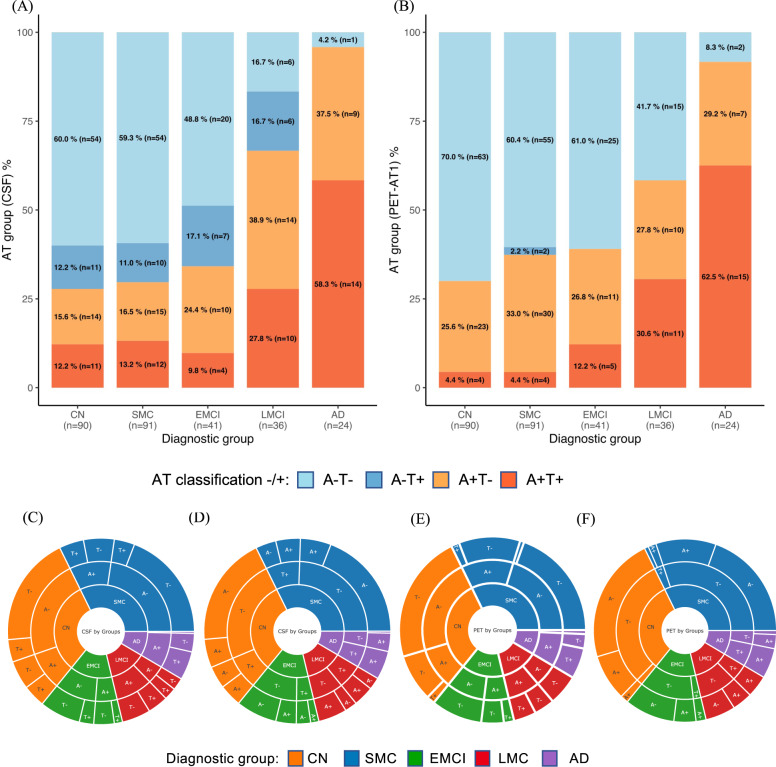
Amyloid-β (A)/tau (T) profiles measured using CSF and imaging biomarkers across diagnostic groups. **A** CSF biomarkers. **B** PET biomarkers. With PET imaging, A+T+ profiles were less prevalent in the CN and SMC groups, and A-T+ profiles were less prevalent in the CN, SMC, EMCI, and LMCI groups, than with CSF. Sunburst graphs with biomarkers visualized in hierarchical fashion. CSF biomarkers, A (Amyloid-β 42) first level (**C**), T (pTau181) first level (**D**). PET biomarkers, A (Amyloid PET) first level (**E**), T (Tau PET) first level (**F**). AD = Alzheimer’s disease; CN = cognitively normal; EMCI = early MCI; LMCI = late MCI; MCI = mild cognitive impairment; SMC = subjective memory concern.

Figure 1a, b shows the proportions of participants with the four AT profiles across the diagnostic groups according to the CSF and imaging biomarkers. There were more A-T+ cases (dark blue) in the CSF panel than in the imaging panel. More specifically, CSF A– cases can be either T+ or T-, while PET T+ cases are usually A+ as also evidenced in Fig. 1c–f. Furthermore, the proportions of A+T+ in the CN and SMC groups were higher (12.2% and 13.2%) in the CSF panel than in the imaging panel (4.4% for both groups).

### Correlations between CSF and imaging ATN biomarkers

**Fig. 2:**
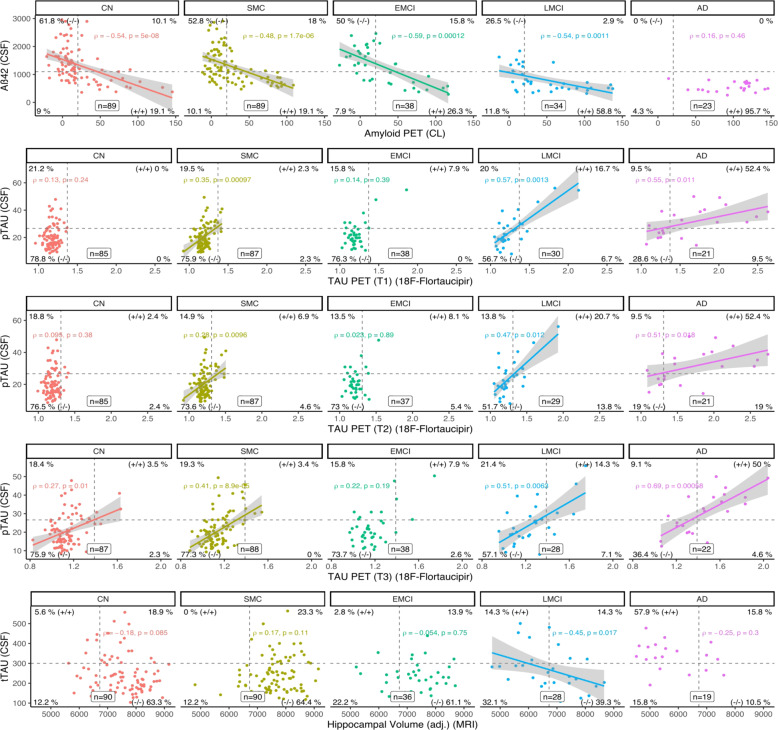
Correlations between CSF and imaging (PET and MRI) biomarkers across diagnostic groups. A = PET amyloid-β ^18^F-florbetapir PET and ^18^F-florbetaben levels; AD = Alzheimer’s disease; CN = cognitively normal; EMCI = early MCI; LMCI = late MCI; MCI = mild cognitive impairment; ROI = region of interest; SMC = subjective memory concern; T = PET tau ^18^F-flortaucipir level; T1 = T measured in the temporal meta-ROI; T2 = T measured in the inferior temporal cortex ROI; T3 = T measured in the entorhinal cortex ROI; N1 = neurodegeneration measured as hippocampal volume adjusted for intracranial volume.

Correlations between the levels of the ATN biomarkers are shown in Fig. 2. Levels of CSF and PET Aβ biomarkers (FBP and FBB, converted in CL) showed moderately to strongly significant associations in all groups (Rho = −0.48 to −0.59) except for the AD group. Conversely, levels of tau biomarkers in CSF (pTau) and imaging (tau PET) showed non-significant correlations in the CN group for T1 and T2 but not for T3 (entorhinal ROI) where the correlation was weak but significant (Rho = 0.27, *p* = 0.01); while in the SMC group, pTau correlated significantly but modestly with tau PET across the ROIs (Rho = 0.28–0.41, *p* < 0.01). As for the CI groups, while the EMCI group showed no significant correlation between pTau and tau PET, LMCI and AD demonstrated moderate to strong significant associations across the PET tau ROIs (Rho = 0.47–0.69, *p* < 0.02). Furthermore, in the EMCI group, CSF T+ and PET T+ (temporal meta-ROI) were 24% and 8%, respectively while, in LMCI, 35 and 23%. This indicates that EMCI and LMCI are two distinct groups with regard to tau pathology. Levels of the neurodegeneration biomarkers in CSF (tTau) and imaging (HVa) did not correlate significantly except in the LMCI group (Rho = −0.45, *p* = 0.017). The lack of associations, especially in the CU groups, demonstrates that tTau and HVa levels are not interchangeable to estimate neurodegeneration. Correlations between additional pairs of biomarkers were also tested in the CU/CI and diagnostic groups (Supplementary Fig. 1).

### Concordance and discordance between CSF and imaging ATN biomarkers

**Fig. 3:**
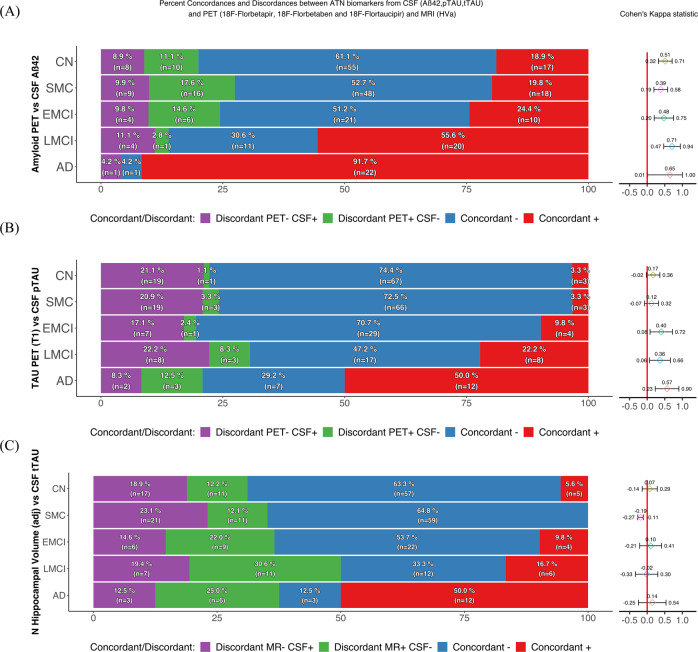
Concordance and discordance between amyloid-β, tau, and neurodegeneration (ATN) biomarkers. Amyloid-β (panel A), tau (panel B), and neurodegeneration (panel C) concordance/discordance profiles between pairs of biomarkers (CSF vs imaging). Cohen’s Kappa statistics allowed numerical comparisons between pairs of profiles obtained using different assessment methods to obtain a coefficient that measured the degree of concordance between the methods. Agreement was defined as coefficient values >0.4 (fair agreement) ranging up to 1 (perfect agreement). Aβ amyloid-β, AD Alzheimer’s disease, CN cognitively normal, EMCI early MCI, HVa hippocampal volume adjusted for intracranial volume, LMCI late MCI, MCI mild cognitive impairment, pTau tau phosphorylated at Thr181, SMC subjective memory concern, tTau total.

The concordance/discordance (as evaluated with Cohen’s Kappa values) of the CSF and imaging biomarkers with regard to the ATN status across diagnostic groups is shown in Fig. 3.

For the Aβ biomarkers (panel A), the agreement was moderate across the diagnostic groups, increasing gradually with increasing cognitive impairment. The LMCI group had the highest concordance, while AD group was affected by the greatest statistical uncertainty due to the lack of PET+/CSF- cases.

In contrast to Aβ, the Cohen’s kappa for Tau status varied across the diagnostic groups. For discordance, isolated CSF pTau positivity was more prevalent than isolated imaging tau positivity. In agreement with these findings, more supporting results on different Tau ROIs and their comparison can be found in Supplementary Fig. 2a and b.

For the neurodegenerative biomarkers, there was no clear pattern of agreement among the diagnostic groups with the exception of the SMC group, where a clear lack of agreement was detected. Concordances between the other combinations of neurodegeneration biomarkers are shown in Supplementary Fig. 2c.

### ATN biomarkers: CSF profiles vs imaging profiles

**Fig. 4:**
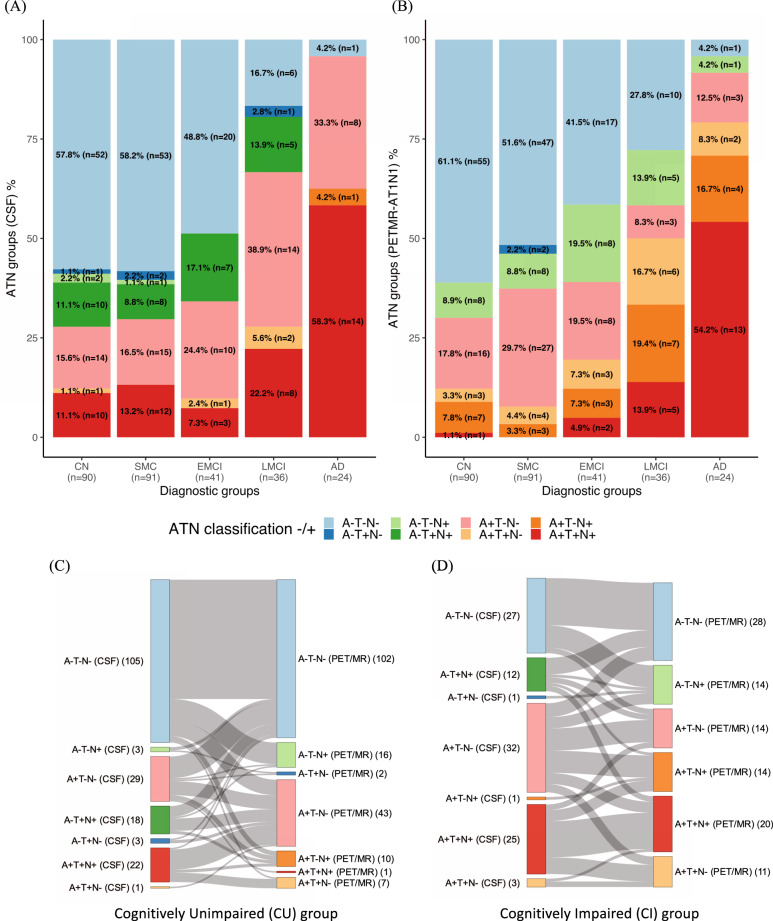
ATN (amyloid-β, tau and neurodegeneration) profiles composed with the six biomarkers, by diagnosis group and cognition. The ATN profiles from the CSF biomarker results (**A**) were different from those from the imaging biomarker results (**B**); Sankey diagrams that represent the correspondence between the CSF and imaging panels in the same subject show disagreement of the ATN classification between the two panels for the CU (**C**) and CI (**D**) groups. AD = Alzheimer’s disease; CN = cognitively normal; EMCI = early MCI; LMCI = late MCI; MCI = mild cognitive impairment; SMC = subjective memory concern.

The ATN profiles resulting from CSF and imaging biomarker results were substantially discordant (see Fig. 4, and Supplementary Figs. 3 and 4a,b). Specifically, the CSF biomarker results indicated a higher prevalence of the A+T+N+ profile than the imaging biomarker results in the CU groups (CN and SMC). In both CU and CI (EMCI, LMCI and AD) groups, disagreement between the tau and neurodegeneration profiles (especially for A+T-N+ and A-T-N + profiles) was more prevalent in the imaging panel than in the CSF panel. These disagreements between the CSF and imaging panels of biomarkers are also visualized in Sankey diagrams for the CU and CI groups, in Fig. 4c, d. The A-T+N+ profile, which was mostly present in the CSF panel for both the CU and CI groups, corresponded generally with a single profile for the imaging panel (A-T-N-). Similar discrepancies between CSF and imaging biomarker results were observed when evaluating the other PET tau regions (T2, T3) and when using cortical thickness as N (N2) (Supplementary Figs. 3 and 4a, b).

### Prediction of cognitive decline by CSF and imaging according to Aβ and tau biomarkers

**Fig. 5:**
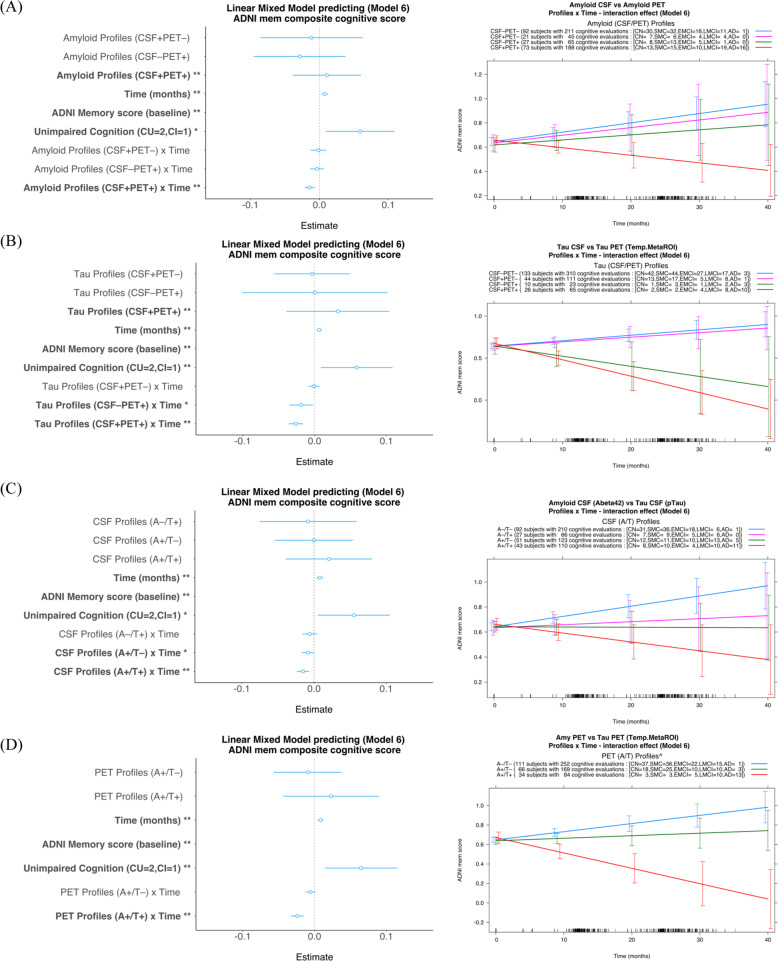
Amyloid-β and tau profiles (CSF and PET together) and CSF and PET profiles (amyloid-β and tau together) tested as predictors for cognitive decline. **A** Among the amyloid-β profiles, only the concordant positive profile (CSF+/PET+) showed a significant negative interaction effect on longitudinal episodic memory decline; **B** Among the tau profiles, only the profiles positive for imaging biomarkers, irrespective of CSF status, (CSF+/PET+, CSF-/PET+) showed a significant negative interaction effect on longitudinal episodic memory decline. **C** CSF profiles: there was a significant interaction between Aβ42 and time (stable or cognitive decline) irrespective of whether pTau was positive or negative; **D** PET profiles: ^The PET profile A-T+ was excluded from the models for this analysis since only one subject had valid data for longitudinal analysis (one more had only baseline measurements). There was a significant interaction between PET tau and time (cognitive decline) only when PET Aβ was positive (A+T+), meaning that PET amyloid-β is predictive only if PET tau is positive and, given that there were two cases (one cross-sectional and one longitudinal case) with PET A-T+ , PET tau is a preferable predictor. *p ≦ 0.05, **p ≦ 0.01. The ADNI_MEM baseline, as expected, had a substantial impact on the model estimations, and its coefficient was consequently about tenfold higher than that of the second highest factor; hence the range selected to display the factor coefficient has been tailored to show differences among the smaller factors. The full representation of the coefficient for each model tested is presented in the SI. AD = Alzheimer’s disease; CN = cognitively normal; EMCI = early MCI; LMCI = late MCI; MCI = mild cognitive impairment; SMC = subjective memory concern.

LMMs were used to test the effect of concordant/discordant biomarker status (CSF vs imaging biomarkers) in predicting prospective cognitive decline, as measured with the composite ADNI_MEM score (*n* = 213 with at least two ADNI_MEM measures). Separate models were applied for the Aβ and tau biomarkers, and ten models were tested (more details in Supplementary Table 2a, b). For both Aβ (CSF/PET) and tau (CSF/PET) analyses, the model with the lowest BIC value among the eight models with comparable datasets was Model 6 (Fig. 5a, b). For tau (CSF/PET) model 6, the Levene test was not significant (assumption not violated), while for Aβ (CSF/PET) model 6, the Levene test was significant (*p* = 0.03), but after a visual inspection of the residuals the assumption was considered not violated. With regard to the Aβ biomarkers, only the concordant positive profile (CSF+/PET+) showed a significant negative interaction effect on episodic memory decline (compared to CSF-/PET-, *p* = 0.0002), while the estimates of the interaction with time for the other profiles were not significant (Fig. 5a).

With regard to the tau biomarkers, only the profiles PET tau-positive, irrespective of CSF status, (CSF+/PET+, CSF-/PET+) showed a significant negative interaction effect with time (relative to CSF-/PET-), suggesting an overall decline in episodic memory in such groups (*p* ≤ 0.02), while the estimate of the interaction with time for the CSF+/PET- profile was not significant (Fig. 5b). Of note, 28% of PET tau-positive cases included in the longitudinal analysis were CSF pTau-negative, revealing that CSF pTau is less accurate than PET tau in predicting cognitive decline in the 38% of cases with low or no cognitive impairment (CN, SMC, EMCI).

### Prediction of cognitive decline by Aβ and tau biomarkers according to CSF and imaging

The same ten models tested in the analyses of the previous section were tested for prediction of cognitive decline from the Aβ/tau profiles according to separate models for CSF and imaging (see more details in Supplementary Table 3a, b). Again, the model with the lowest BIC value among the eight models with comparable datasets was Model 6 (Fig. 5c, d). For both CSF and imaging model 6, the Levene test was not significant (assumption not violated). With regard to CSF biomarkers, Aβ positivity (A+T + or A+T-) had a negative effect on cognition relative to the control profile A-T- (*p* ≤ 0.05), irrespective of tau status, while the effects of tau positivity and Aβ negativity (A-T+) did not reach statistical significance. The A-T- profile had a generally positive effect on episodic memory, probably as a result of the learning effect of repeated testing (Fig. 5c).

With regard to the imaging biomarkers, tau positivity (A+T+) had a negative effect on longitudinal episodic memory performance cognition relative to the control profile A-T- (*p* < 0.001), while the A+T- profile did not show this effect (Fig. 5d). The A-T+ group had only two members with available longitudinal data (both borderline case according to the cutoff points used for Amyloid PET and one also for Tau PET biomarkers) and they were therefore not investigated in the models.

### Prediction of cognitive decline with N biomarkers as co-variate

The predictive power of neurodegeneration biomarkers was evaluated in Models 8, 9 and 10. Model 8 (Supplementary Figs. 5a, b, and 6 a, b) had the best BIC value (Supplementary Tables 2a,b and 3a,b) and, although they were not comparable due to different sample size, this suggests that HVa assessment could be a valuable complement to the model for predicting cognitive decline.

## Discussion

The main findings of this study are: (a) The CSF and imaging biomarkers resulted in differential ATN profiles, mainly as a result of discordance in the biomarker modalities between the tau and neurodegeneration components; (b) PET tau positivity was a better predictor of short-term cognitive decline than PET Aβ or CSF pTau results.

In this study, we started with the diagnosis (based on purely clinical criteria independent of biomarker assessments) and then profiled the pathological burden of the participants from CSF and imaging biomarkers separately, using the ATN profiling system. This approach was used to evaluate concordance between the CSF and imaging biomarkers impartially for the different diagnostic groups and to improve understanding of which biomarker, especially where there was discordance, might provide better accuracy for predicting cognitive decline.

We investigated the associations and concordance between ATN biomarkers, with special attention to comparisons of pairs within the same ATN category: CSF Aβ42 vs PET Aβ, CSF pTau vs PET tau and CSF tTau vs MRI-derived neurodegeneration biomarkers. We found that Aβ results correlated more closely between CSF and PET than other biomarker pairs, and agreed across the different diagnostic groups, in line with other reports [2, 22]. Interestingly, in the cases of discordance and isolated CSF or PET Aβ positivity, there was no short-term decline in cognition. This suggests that the two modalities could track slightly different aspects of Aβ accumulation, as previously suggested [23], but neither showed superiority in terms of prediction accuracy in our sample.

We found relative disagreement between CSF pTau and PET tau results, and even more disagreement between tTau and MRI-based neurodegeneration biomarkers, which indicates the lack of interchangeability between CSF and imaging modalities for monitoring tau accumulation and neurodegeneration. The higher range of values for CSF pTau and the higher proportion of participants across diagnostic groups with T+, relative to PET, points to earlier changes in CSF tau measures in AD spectrum, as previously suggested [24]. However, our longitudinal analyses did not support the idea that early changes in CSF values are related to a short-term cognitive decline, questioning the prognostic value of CSF biomarkers. As previously speculated [25], CSF pTau could be seen as a snapshot of tau accumulation and is not automatically related to the brain tau burden, as shown in early CSF vs autopsy studies [26, 27]. In contrast, the presence of pathological levels of tau in the brain when measured with PET tau imaging predicted a steeper decline in cognition longitudinally, irrespective of the CSF results. This provides evidence of the superiority of PET tau measurement over CSF tau measurement for providing an accurate prognosis and for recruitment of individuals for clinical trials early in the disease continuum.

The studied neurodegenerative biomarkers, more than other pairs of considered biomarkers, seemed to not be interchangeable. This lack of agreement was present across all diagnostic groups and also within the different MRI measures (HV vs cortical thickness), with no clear agreement pattern across the different diagnostic groups. Even if CSF total Tau is not the best neurodegeneration biomarkers, compared to plasma NfL (for example) [28], CSF tTau could be considered to reflect the amount of neuronal damage at a given time point, brain atrophy (even as a cross-sectional time point) reflects the natural history of neurodegeneration [29]. The discordance between the two MRI measures could be explained by a previous study which showed how cortical thinning and hippocampal volume decrease have different acceleration slopes [30]. Accordingly, we found the highest concordance between MRI measures in the EMCI group, which represents an intermediate stage between preclinical AD and the dementia stage of the disease.

The ATN profiling confirmed the lack of interchangeability observed in the other analyses between CSF and imaging modalities; the discordance between the two should be taken into account in future guidelines for the in vivo characterization of AD-related pathological changes using biomarkers, instead of the existing algorithm [1]. This is supported by the concordance analyses in a recent publication regarding another sample from the BIOFINDER study [2].

When looking at the pairs of neuropathological markers (Aβ and tau together) within each modality for defining the biomarkers most predictive of cognitive decline, different results were seen for CSF and imaging biomarkers. For CSF, Aβ positivity was related to worse cognition longitudinally, irrespective of tau status, although the estimates for this effect were small. For imaging, rapid cognitive decline was predicted by PET tau positivity rather than PET Aβ positivity, since tau positivity was coupled with Aβ positivity but not vice versa. It is known that Aβ positivity per se does not predict short-term cognitive decline, given the age-specific positivity rate of >50% for people aged 80–90 years [31] and given that only a subset of CI PET Aβ-positive patients declines over time (low prognostic specificity) [32]. Our evidence suggests that PET tau imaging should be prioritized over other markers (CSF sampling or PET Aβ) in the clinical assessment of cognitive impairment.

Another issue to consider is the selection of which tau region to use for assessing cognitive decline, since some brain regions (e.g., the entorhinal cortex) are more affected in the early stages of the disease and might progress slower than others; while others are more affected at the AD stage (e.g., the temporal lobe) [33]. Our data confirm this, demonstrating a difference between the entorhinal ROI and the two temporal ROIs and extend previous knowledge to the notion of differences between SMC and CN groups.

### Limitations

The follow-up was relatively short (up to 3 years). Despite the large cohort of subjects to choose from, we limited the patient selection to those for whom we had results for all six ATN biomarkers, which limited interpretation of the results and requires validation in other samples. We focused on the dichotomization (more clinically oriented) and did not investigate the biomarkers as continuous variables. The choice of total Tau as fluid biomarker for neurodegeneration was forced due to the availability of the ADNI data, a more suitable biomarker such as plasma NfL would have been preferrable but was not available for ADNI3 cohort.

### Conclusions

From these data, it can be concluded that CSF and imaging biomarkers differ considerably within the ATN framework; the most effective of the investigated biomarkers for predicting cognitive decline was the PET tau biomarker, alone. Our results provide support for the prioritization of PET tau over other biomarkers in the assessment of patients with cognitive impairment; if the result is then positive, there is a high chance of rapid cognitive decline. In the presence of a PET tau-positive result, Aβ testing might not be necessary since it is likely that PET or CSF Aβ would be positive too, while CSF tau would show low specificity for detecting short term AD-related cognitive decline. If PET tau is negative, it is suggested that there will not be cognitive decline in the short term (up to 3 years of follow-up time) and other investigations with long-term biomarkers could then be considered.

## Supplementary information

Supplemental Material
