# Supplementary material for: Alzheimer’s disease profiled by fluid and imaging markers: tau PET best predicts cognitive decline
Source: Mol Psychiatry. 2021 Oct 1;26(10):5888–98. doi: 10.1038/s41380-021-01263-2 (PMC8758489; doi:10.1038/s41380-021-01263-2)

Supplementary Information – Methods and Results

**Additional methods:**

Supplementary Fig 2, 3a, b and c show correlations between pairs of biomarkers. The correlations have been verified also via exclusion of outliers via the Cook’s distance method (4/n, standard threshold). Supplementary Fig 2 present amyloid PET data aggregated via Centiloid (CL) conversion, validated and published by ADNI (http://adni.loni.usc.edu/wp-content/themes/freshnews-dev-v2/documents/pet/ADNI%20Centiloids%20Final.pdf).

**Results:**

1. ***Sample characteristics***

Supplementary Table 1. ATN Biomarkers by Diagnosis


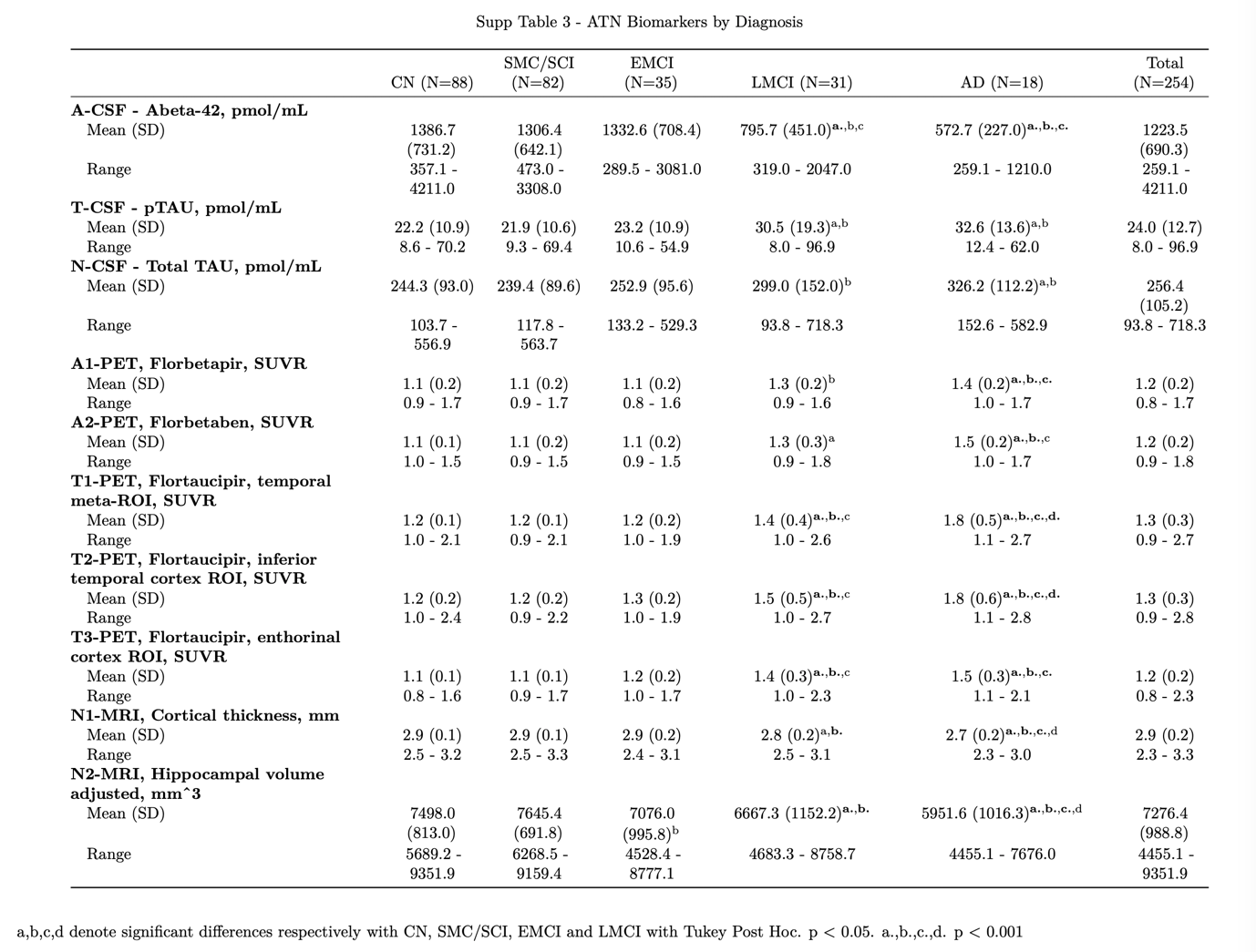


1. ***Correlations between CSF and imaging ATN biomarkers***

Supplementary Fig 1. Correlations between CSF and Imaging biomarkers according to cognitive impairment


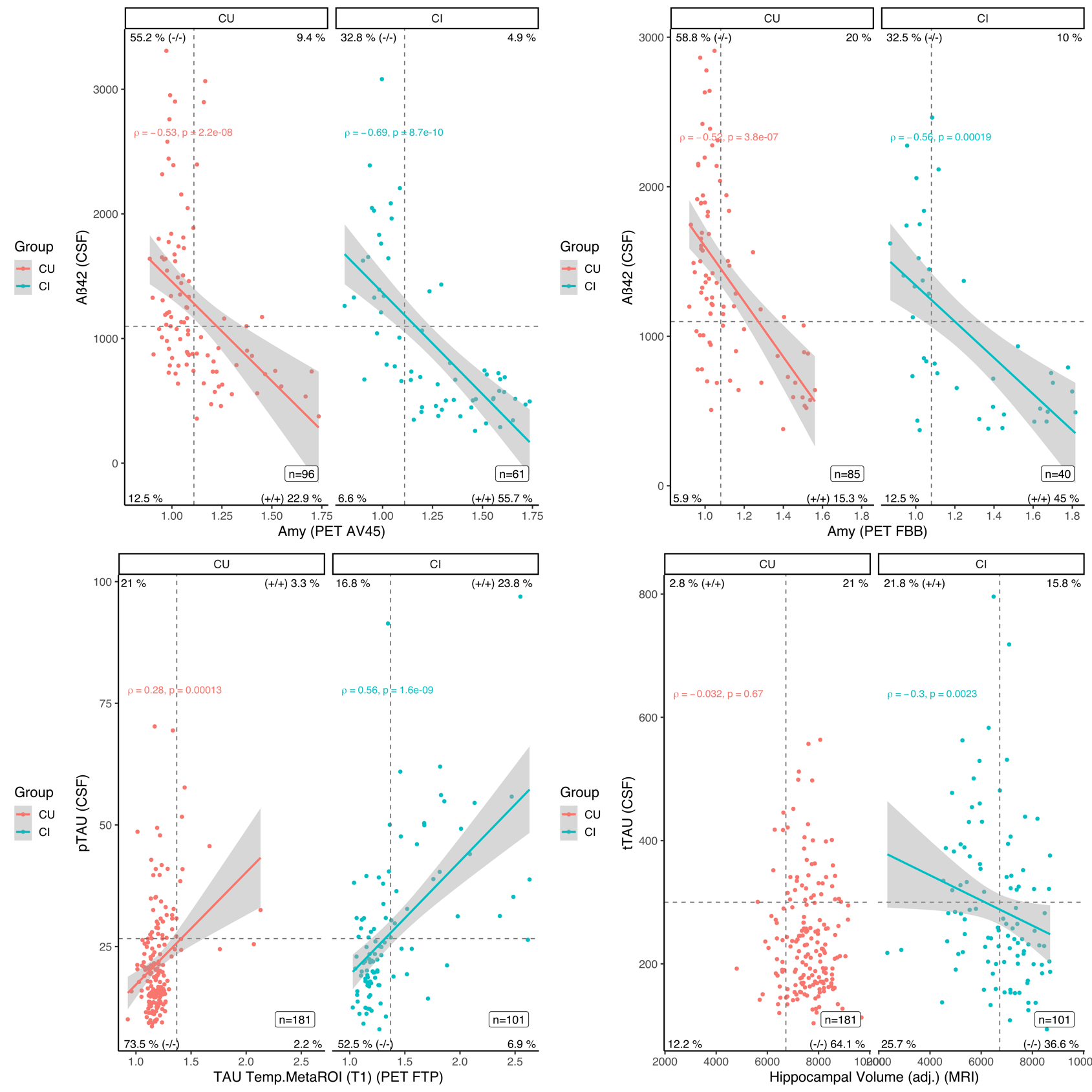


1. ***Concordance and discordance between CSF and imaging ATN biomarkers***

Supplementary Fig 2a. Percentual of concordances and discordances between TAU CSF (pTAU) and TAU PET (AV1451) biomarkers (three different ROIs)


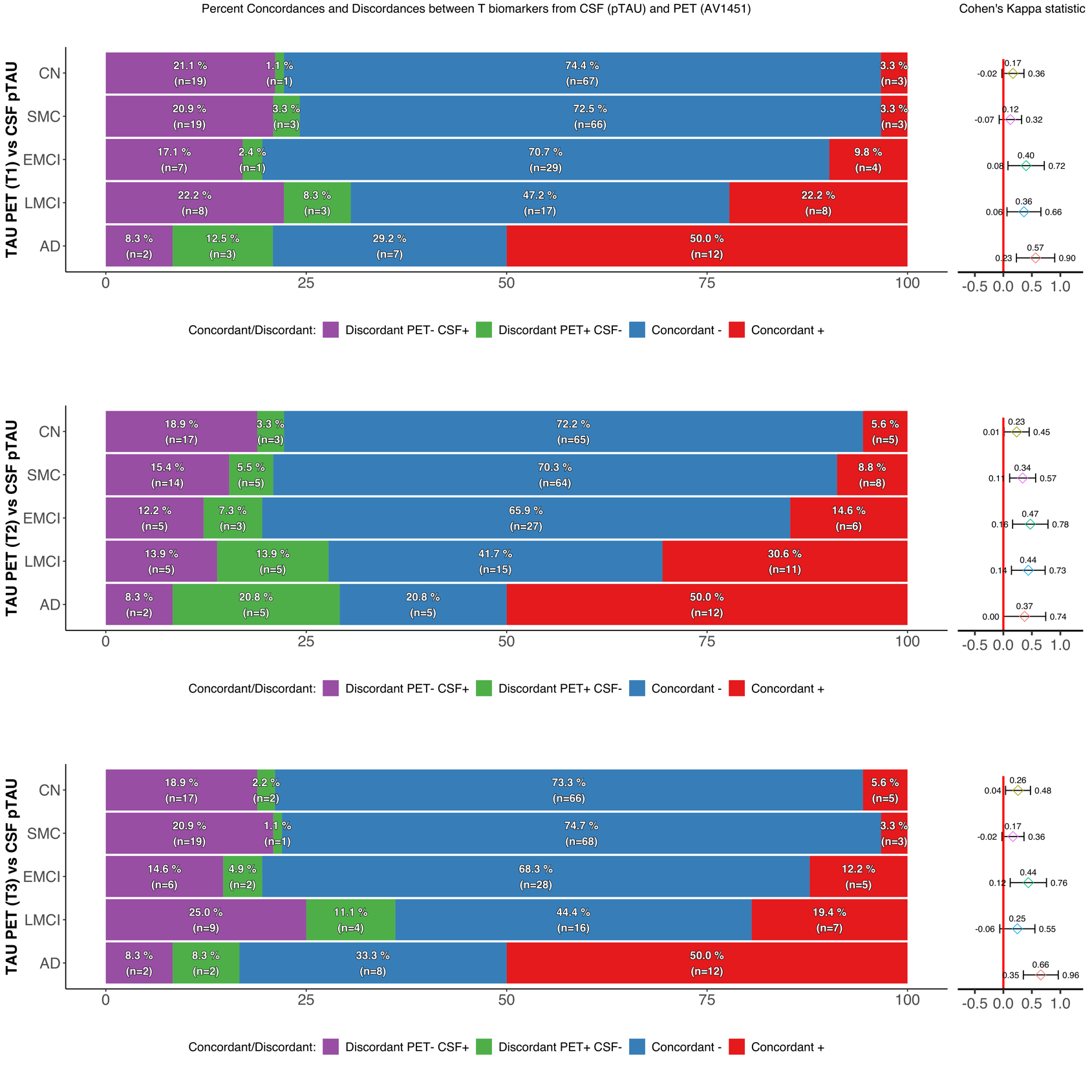


Supplementary Fig 2b. Percentual of concordances and discordances between TAU PET (AV1451) ROIs


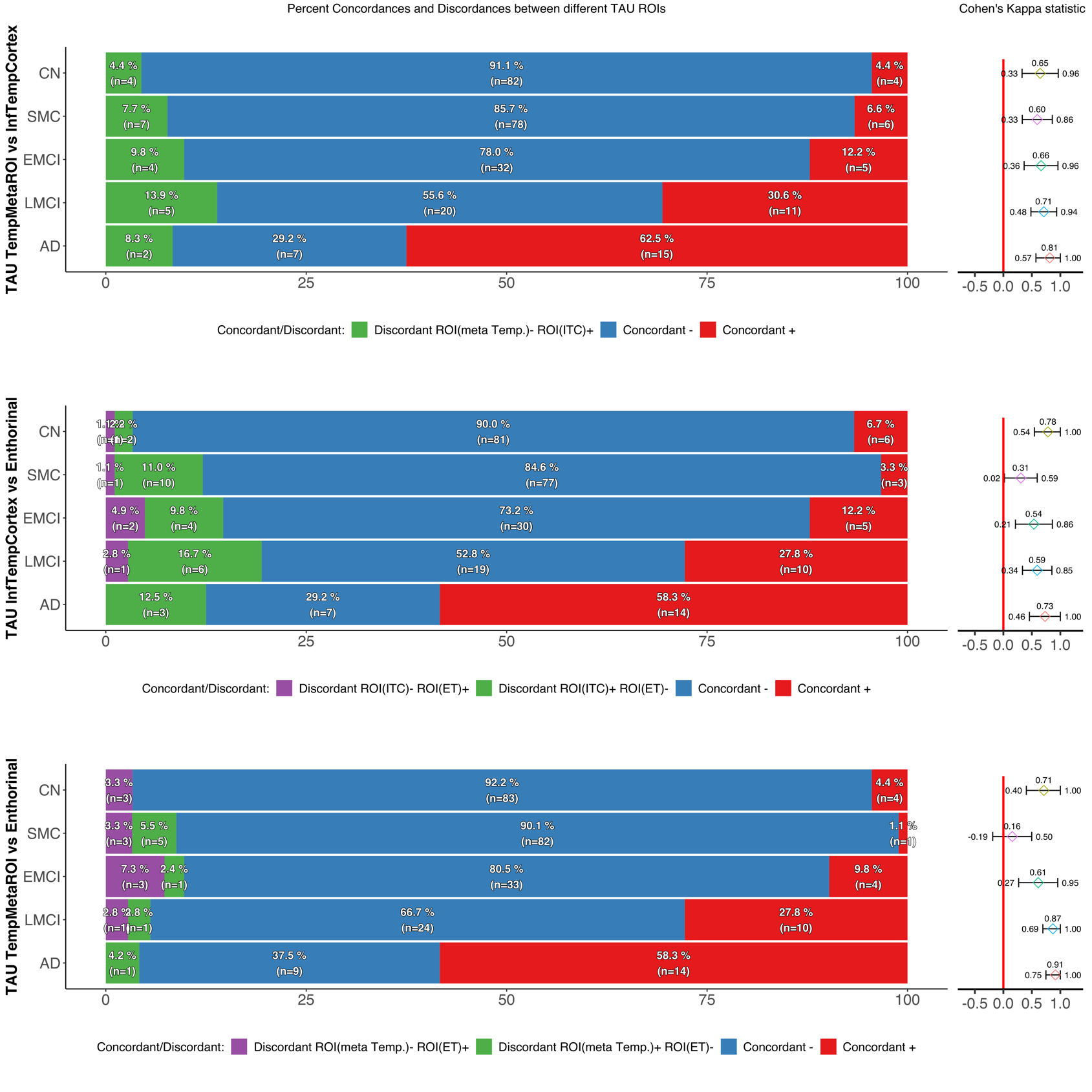


Supplementary Fig 2c. Percentual of concordances and discordances between N MR (Cortical thickness and Hippocampal volume adj.) and N CSF (tTAU)


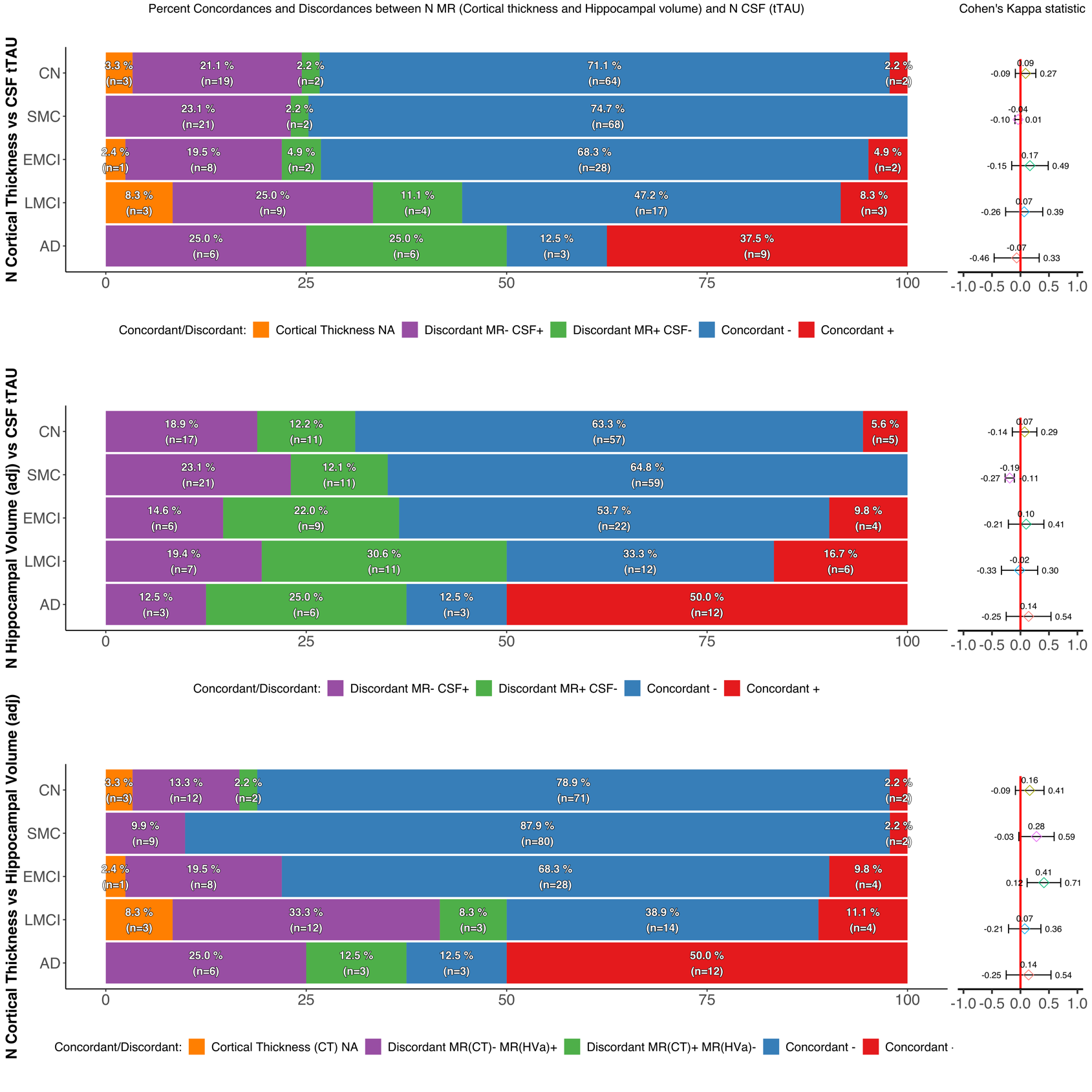


1. ***ATN biomarkers: CSF profiles vs imaging profiles***

Supplementary Fig 3. ATN profiles composed with the 6 different biomarkers (3 CSF, A-PET, T-PET, N-HVa-MR)

by cognitive impairment


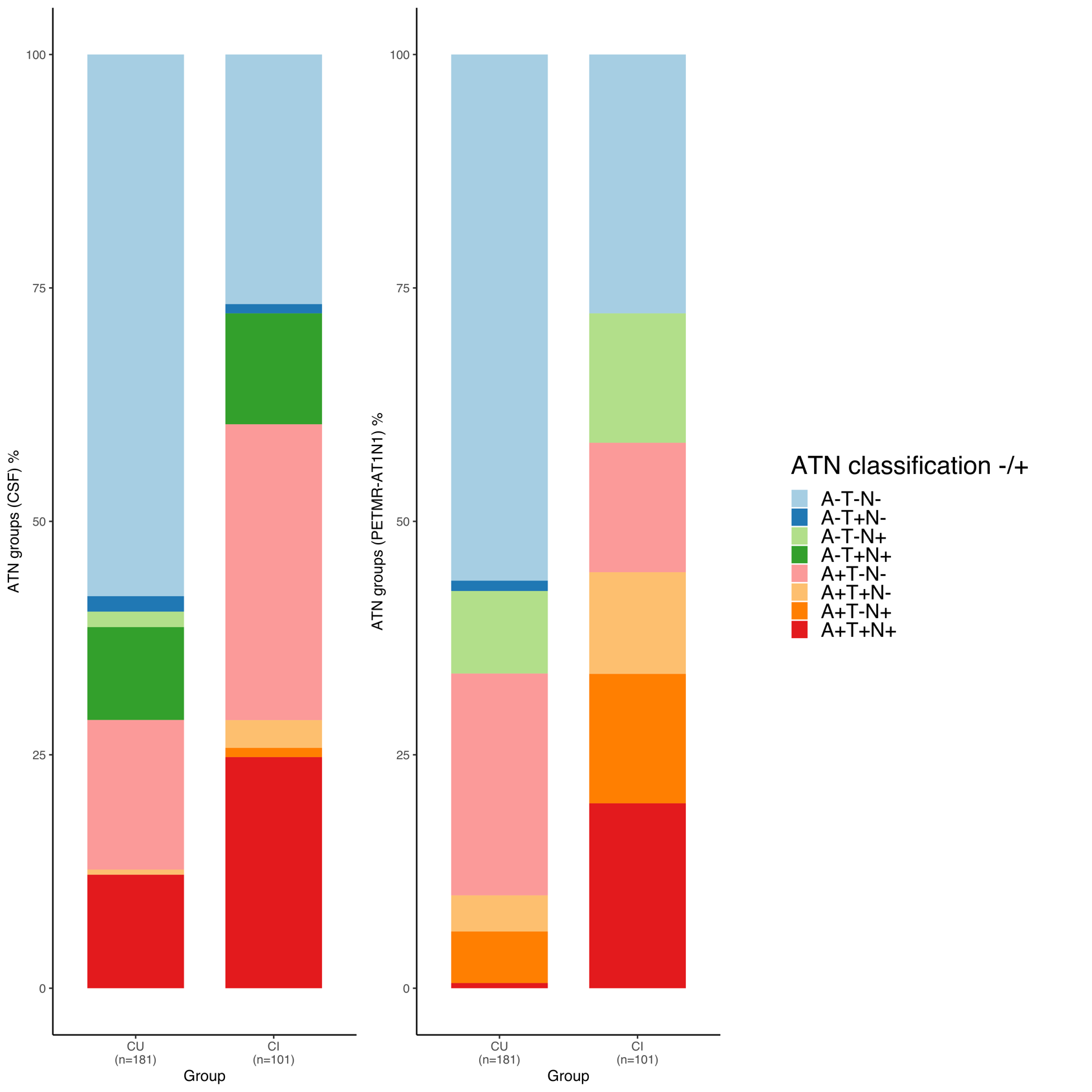


Supplementary Fig 4a. ATN profiles composed with the 6 different combinations of imaging biomarkers by diagnosis


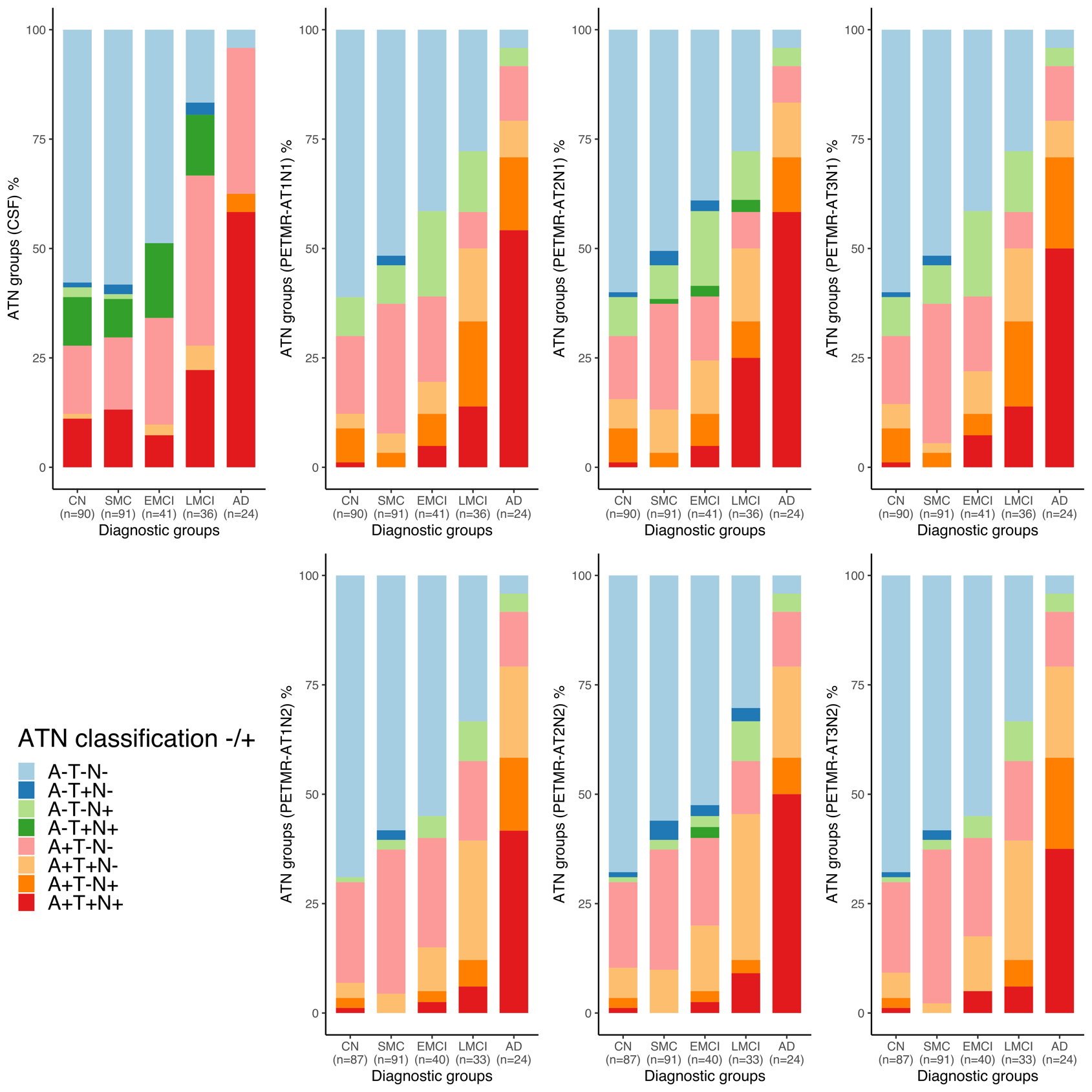


Abbreviations: T1(Tau-PET-Temporal MetaROI), T2(Tau-PET-Inferior Temporal Cortex), T3(Tau-PET-Entorhinal Cortex), (N1-MRI) Hippocampal Volume adjusted, (N2-MRI) Cortical thickness.

Supplementary Fig 4b. ATN profiles composed with the 6 different combinations of imaging biomarkers by cognitive impairment


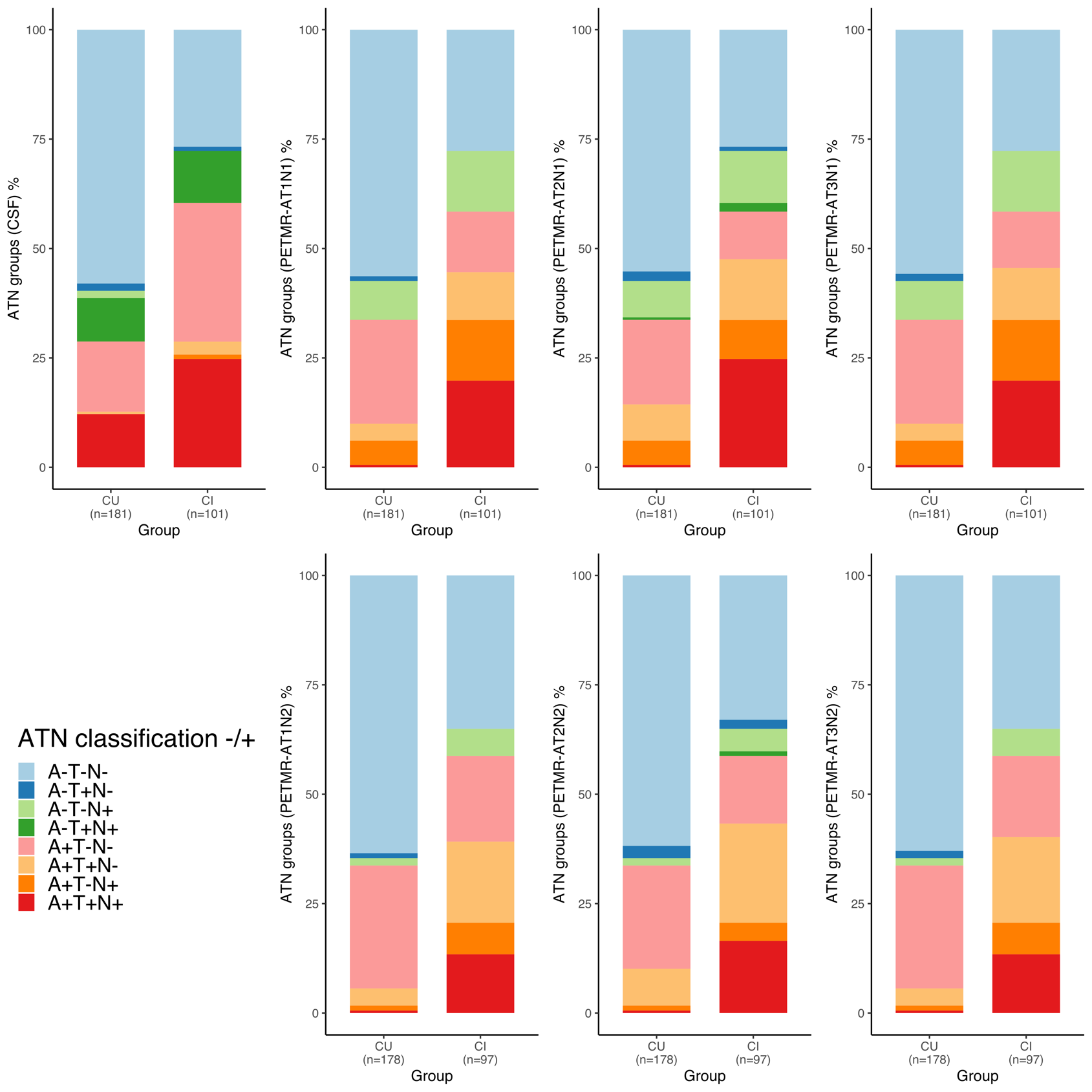


**LMM additional methods:**

The following baseline variables were added as covariates: age, gender, cognitive impairment group (cognitively unimpaired (CU), which included CN and SMC; or cognitively impaired (CI), which included EMCI, LMCI and AD), education, and ApoE4 homozygous genotype. Importantly, the interaction between the profile and time was included in the models. In the tested models, the slope was random, and the intercept fixed but a baseline ADNI_MEM factor was introduced in the equations. The models were also tested by adding ApoE4 carrier and ApoE2 carrier status (Yes/No), separately, but this did not result in significant predictors (data not shown).

Ten models with different combinations of variables were systematically tested and the model with the lowest Schwarz’s Bayesian Information Criterion (BIC) was chosen as the best model.

Models 1-7 and 10 were compared separately from Models 8 and 9 because they included different datasets. Models 8 and 9 included MRI biomarkers at baseline to assess whether the contribution of neurodegenerative measures at baseline would improve the prediction of cognitive change; these models had a smaller dataset (n=136) because the MRI neurodegeneration biomarkers were not acquired at the same baseline visit as the other biomarkers. Variance Influence Factors (VIF) were computed and adjusted for degree of freedom factors (GVIF) and for of the models 6 and 8 resulted less or equal than 1.5, way below the conservative threshold of 5 for multi-collinearity (Stein) that would warrant for centering of the variables.

**Model equations: (**Profiles= Amyloid_Profiles or Tau_Profiles (CSF-PET-, CSF-PET+,PET+CSF-,CSF+PET+)

Model 1 = lmer(ADNI_Memory_cscore ~ Profiles* time_months + ADNI_Memory_cscore_baseline + ApoE4_homo + (0 + time_months | RID) + 1, data=longitudinal_data)

Model 2: lmer(ADNI_Memory_cscore ~ Profiles* time_months + ADNI_Memory_cscore_baseline + ApoE4_homo + Impaired_cognition + (0 + time_months | RID) + 1, data=longitudinal_data)

Model 3: lmer(ADNI_Memory_cscore ~ Profiles* time_months + ADNI_Memory_cscore_baseline + ApoE4_homo + Impaired_cognition + Age_y + (0 + time_months | RID) + 1, data=longitudinal_data)

Model 4: lmer(ADNI_Memory_cscore ~ Profiles* time_months + ADNI_Memory_cscore_baseline + ApoE4_homo + Impaired_cognition + Age_y + Gender_F + (0 + time_months | RID) + 1, data=longitudinal_data)

Model 5: lmer(ADNI_Memory_cscore ~ Profiles* time_months + ADNI_Memory_cscore_baseline + ApoE4_homo + Impaired_cognition + Age_y + Gender_F + Education_y + (0 + time_months | RID) + 1, data=longitudinal_data)

Model 6: lmer(ADNI_Memory_cscore ~ Profiles* time_months + ADNI_Memory_cscore_baseline + Impaired_cognition + (0 + time_months | RID) + 1, data=longitudinal_data)

Model 7: lmer(ADNI_Memory_cscore ~ Profiles* time_months + ADNI_Memory_cscore_baseline + Impaired_cognition + Age_y + (0 + time_months | RID) + 1, data=longitudinal_data)

Model 8: lmer(ADNI_Memory_cscore ~ Profiles* time_months + ADNI_Memory_cscore_baseline + HVa_mm3 + (0 + time_months | RID) + 1, data=longitudinal_data)

Model 9: lmer(ADNI_Memory_cscore ~ Profiles* time_months + ADNI_Memory_cscore_baseline + CorticalThickness_mm + (0 + time_months | RID) + 1, data=longitudinal_data)

Model 10: lmer(ADNI_Memory_cscore ~ Profiles* time_months + ADNI_Memory_cscore_baseline + tTau_pmol_ml + (0 + time_months | RID) + 1, data=longitudinal_data)

1. ***Prediction of cognitive decline by CSF and imaging according to Aβ and tau biomarkers***

Supplementary Table 2a (in relation to Fig 3a of the main text). Amyloid CSF/PET Model comparison


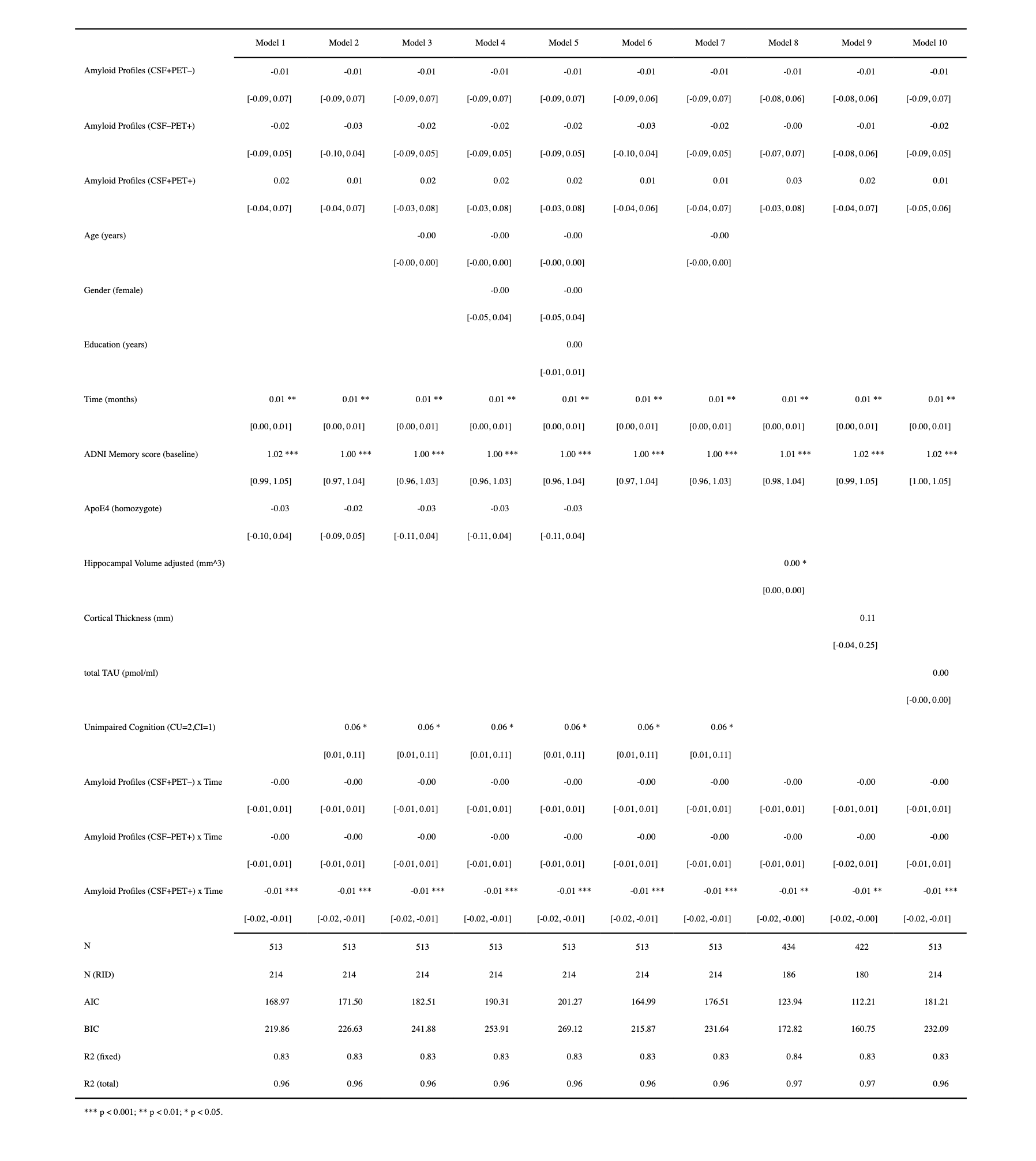


Supplementary Table 2b (in relation to Fig 3b of the main text). Tau CSF/PET Model comparison


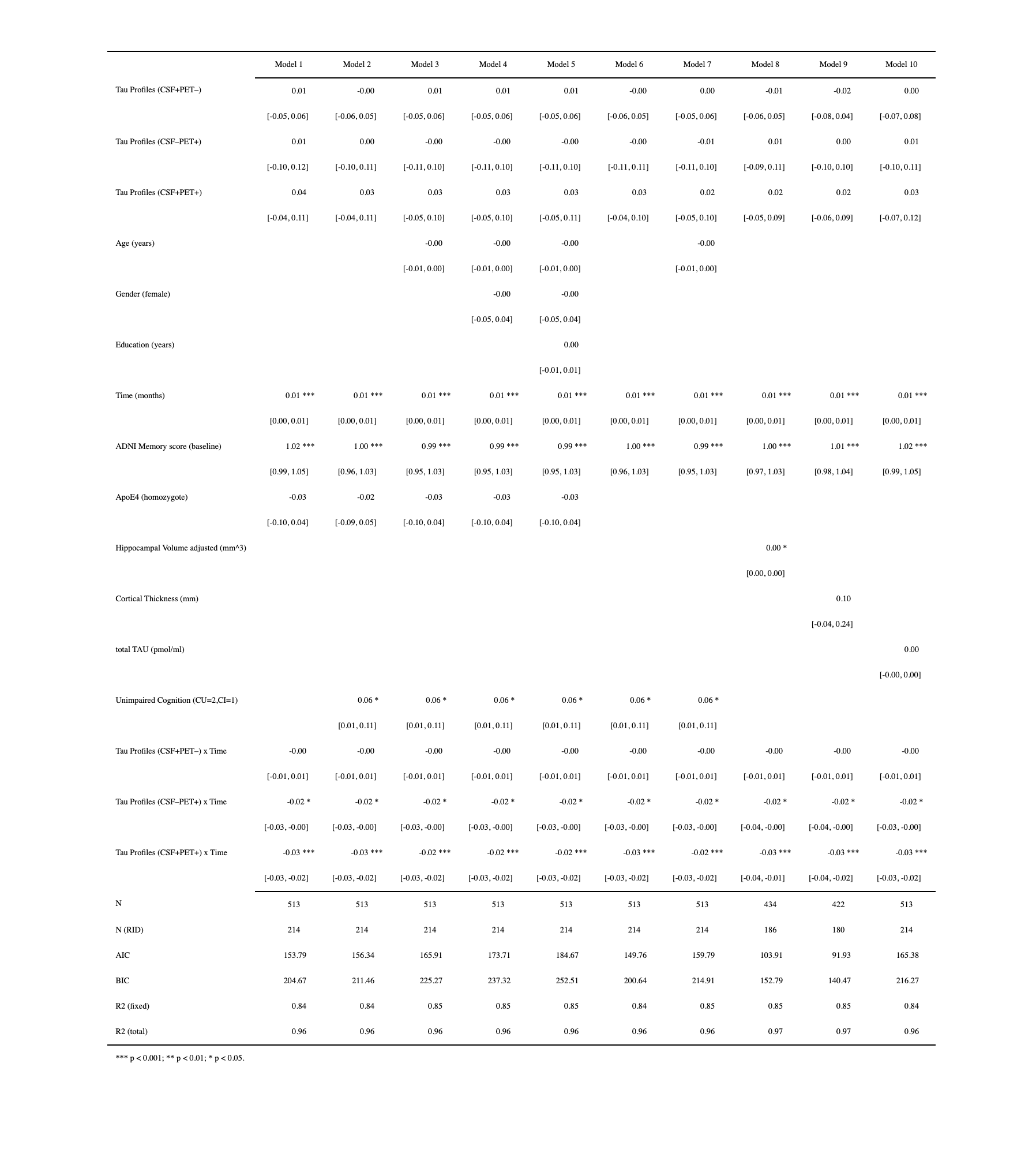


1. ***Prediction of cognitive decline by Aβ and tau biomarkers according to CSF and imaging***

Supplementary Table 3a (in relation to Fig 4a of the main text). CSF (A/T) Model comparison


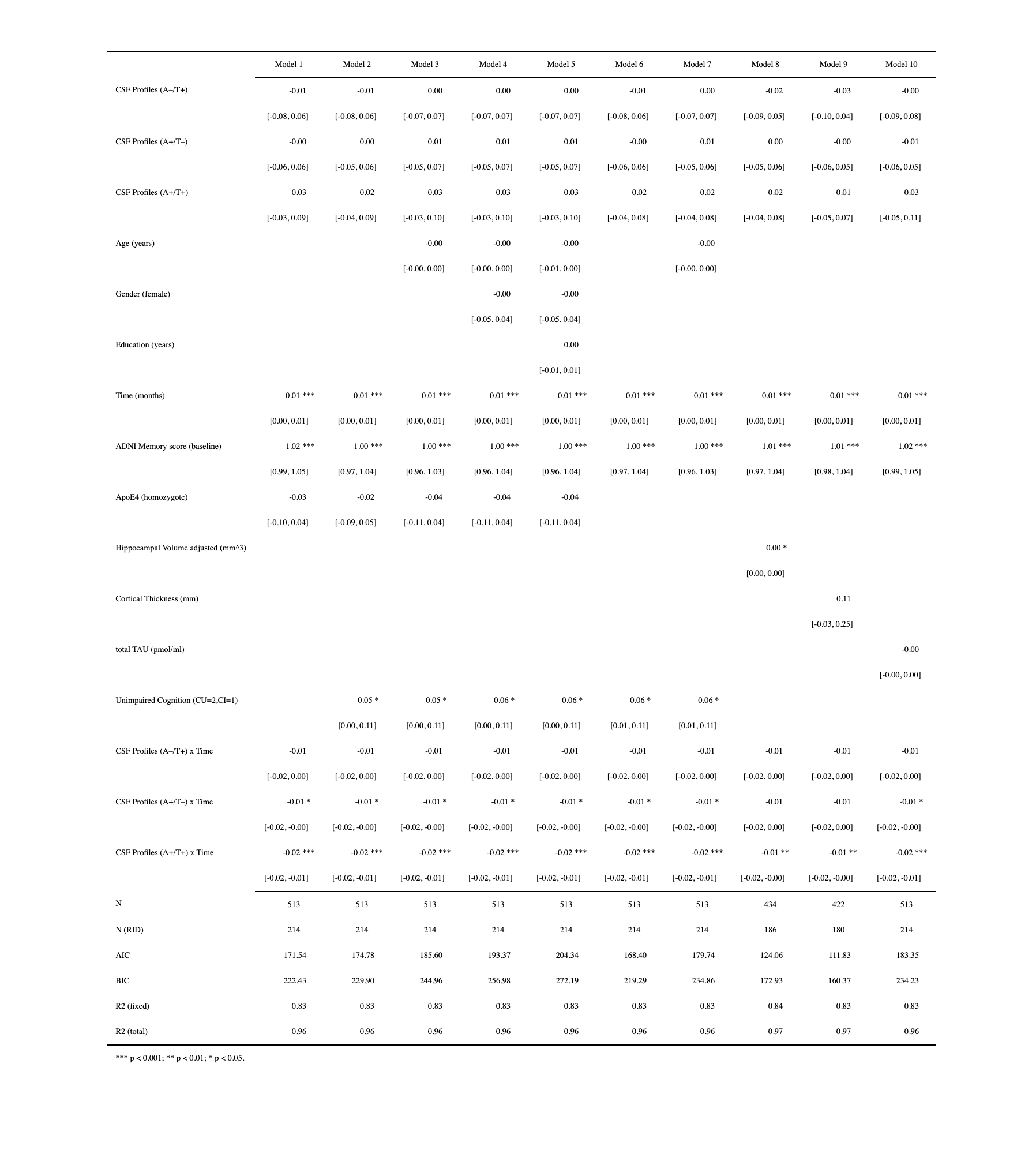


Supplementary Table 3b (in relation to Fig 4b of the main text). PET (A/T) Model comparison


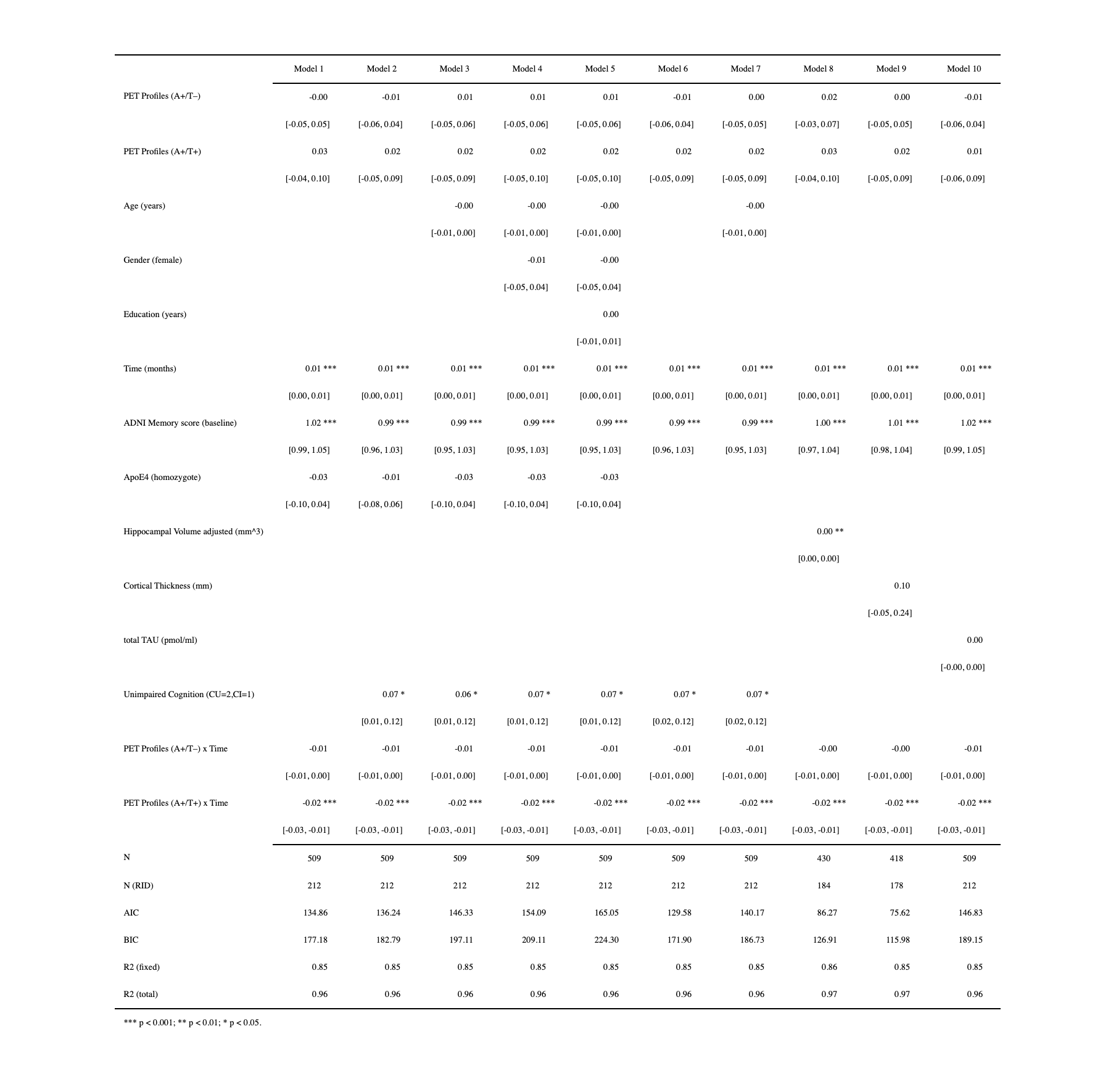


Fig 5a. Amyloid profiles (CSF and PET together) tested as predictors for cognitive decline (Model 8)


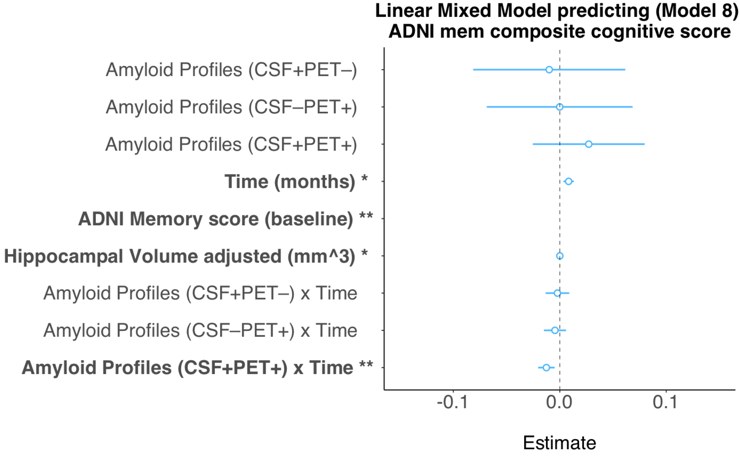

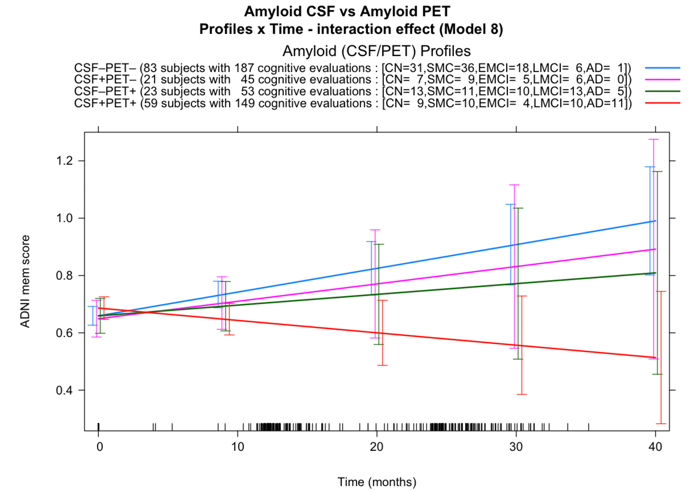


Fig 5b. Tau profiles (CSF and PET together) tested as predictors for cognitive decline (Model 8)


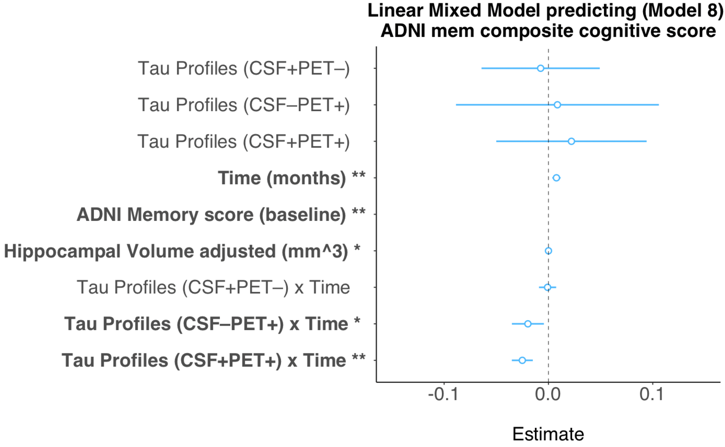

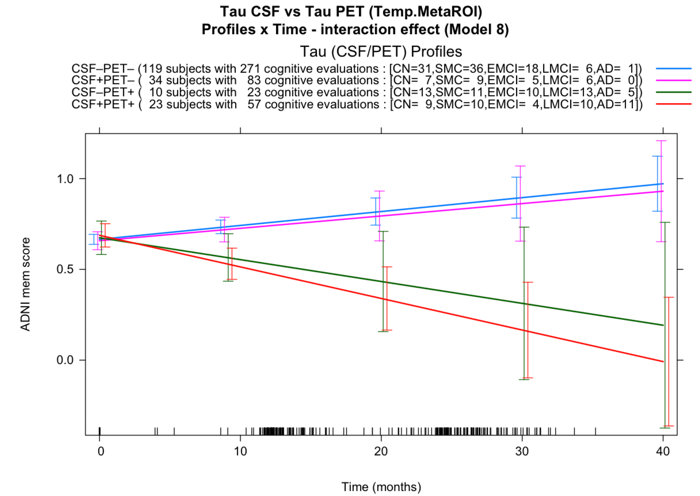


Fig 6a. Amyloid and Tau profiles (CSF only) tested as predictors for cognitive decline (Model 8)


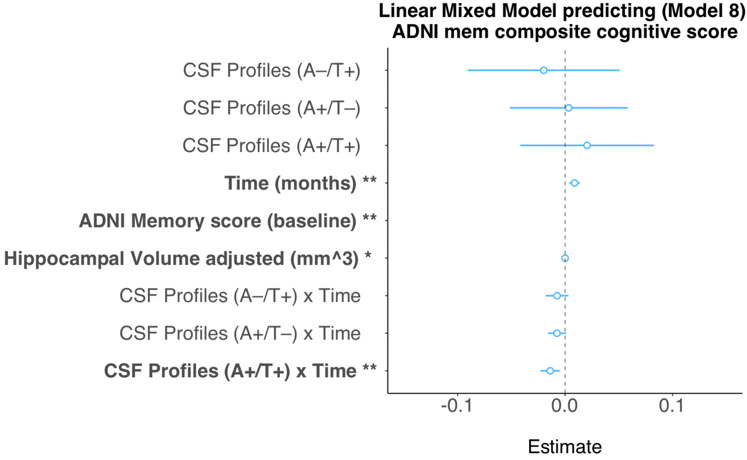

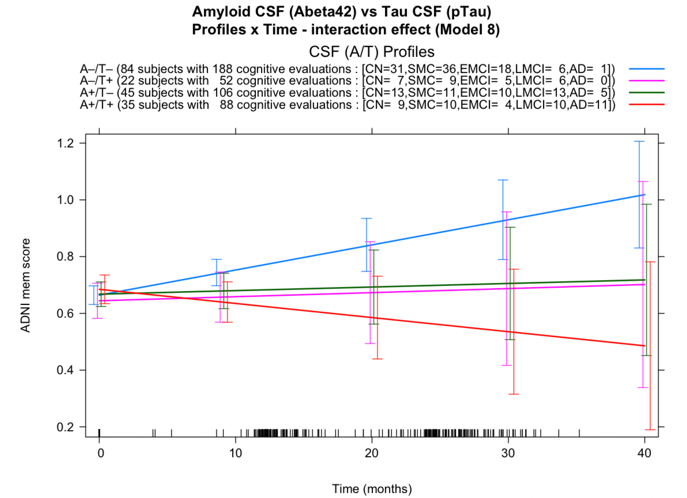


Fig 6b. Amyloid and Tau profiles (PET only) tested as predictors for cognitive decline (Model 8)


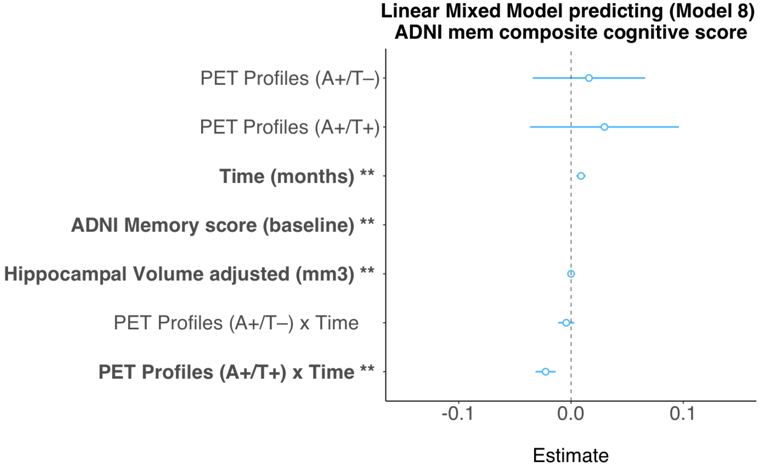

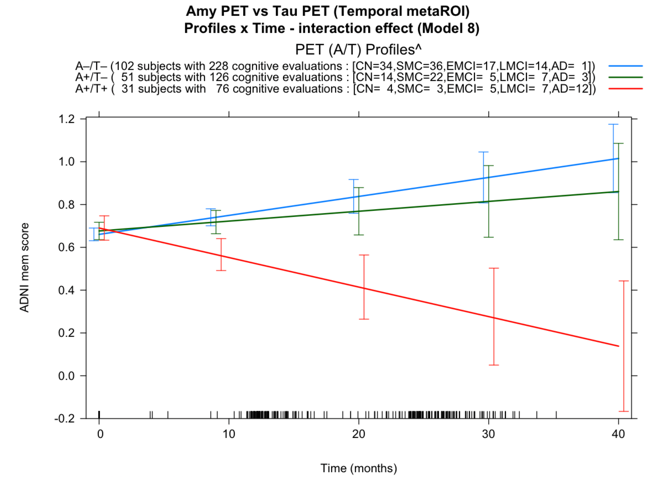

Supplement: Supplementary file 1 — Supplemental Material [file 41380_2021_1263_MOESM1_ESM.docx]
